# Preliminary Results of the *in Vivo* and *in Vitro* Characterization of a Tentacle Venom Fraction from the Jellyfish *Aurelia aurita*

**DOI:** 10.3390/toxins5122420

**Published:** 2013-12-06

**Authors:** Dalia Ponce, Estuardo López-Vera, Manuel B. Aguilar, Judith Sánchez-Rodríguez

**Affiliations:** 1 Instituto de Ciencias del Mar y Limnología, Unidad Académica Puerto Morelos, Universidad Nacional Autónoma de México,77500 Cancún, Quintana Roo, Mexico; E-Mail: daliappg@gmail.com; 2 Instituto de Ciencias del Mar y Limnología, Universidad Nacional Autónoma de México, Circuito Exterior s/n, Ciudad Universitaria, 04510 Coyoacán, Distrito Federal, Mexico; 3 Laboratorio de Neurofarmacología Marina, Departamento de Neurobiología Celular y Molecular, Instituto de Neurobiología, Universidad Nacional Autónoma de México, Campus Juriquilla, Juriquilla 76230, Querétaro, Mexico; E-Mail: maguilar@unam.mx

**Keywords:** *Aurelia aurita*, jellyfish, neurotoxins, nicotinic acetylcholine receptors, Scyphozoa

## Abstract

The neurotoxic effects produced by a tentacle venom extract and a fraction were analyzed and correlated by *in vivo* and *in vitro* approaches. The tentacle venom extract exhibited a wide range of protein components (from 24 to >225 kDa) and produced tetanic reactions, flaccid paralysis, and death when injected into crabs. Two chromatography fractions also produced uncontrolled appendix movements and leg stretching. Further electrophysiological characterization demonstrated that one of these fractions potently inhibited ACh-elicited currents mediated by both vertebrate fetal and adult muscle nicotinic acetylcholine receptors (nAChR) subtypes. Receptor inhibition was concentration-dependent and completely reversible. The calculated IC_50_ values were 1.77 μg/μL for fetal and 2.28 μg/μL for adult muscle nAChRs. The bioactive fraction was composed of a major protein component at ~90 kDa and lacked phospholipase A activity. This work represents the first insight into the interaction of jellyfish venom components and muscle nicotinic receptors.

## 1. Introduction

Scyphozoan jellyfish produce venoms that possess potent biological activities [1]. Like all cnidarians, scyphozoans store and release these bioactive mixtures through specialized organelles known as nematocysts which are highly abundant in the fishing tentacles [2]. Nematocyst venom is mainly involved in effective prey capture and deterrence of predators by the disruption of key physiological processes. Several studies have demonstrated the pharmacological effects of jellyfish toxins when tested in different *in vivo* and *in vitro* preparations [3,4].

The moon jellyfish—*Aurelia aurita*—is a scyphozoan jellyfish with a cosmopolitan distribution often considered harmless to humans [5]. However, significant envenomation has been described for its stings, characterized by local pain, intense itching, ulceration, and appearance of vesiculopapular erythematous eruptions [6,7]. *A. aurita* crude venom and partially purified fractions have been reported to produce hemolysis, cytotoxicity, dermonecrosis, lethality, neurotoxicity, and to contain phospholipase activity [7,8,9,10,11,12]. In particular, only a few studies have focused on characterizing *A. aurita* neurotoxins. In this regard, Segura-Puertas *et al.* [7] reported some protein fractions that caused paralysis and death of crabs when tested *in vivo*. Likewise, Kihara and colleagues [10] demonstrated that a venom preparation caused depolarization of frog muscle membranes that was attributed mainly to sodium ion influx. A similar feature has been suggested for other scyphozoan jellyfish venoms that appear to induce the opening or activation of non-selective cationic channels and a subsequent increase of inward sodium-elicited currents [13,14,15,16]. However, the identification of the specific molecular targets involved in the mechanism of action of scyphozoan neurotoxins remains unclear.

Nicotinic acetylcholine receptors (nAChRs) are non-selective cation channels known to be affected by a wide variety of neurotoxins [17]. The vertebrate muscle nAChRs are divided into fetal (α1β1γδ) and adult (α1β1εδ) subtypes depending on the subunit stoichiometry and the developmental stage of muscle [18]. Both subtypes are found at the postsynaptic membrane of the neuromuscular junction and their inhibition has been demonstrated to lead to skeletal muscle paralysis [19]. Muscle nAChRs are targeted by several neurotoxins produced by *Elapid* and *Hydrophiid* snakes, and marine *Conus* snails. Snake curaremimetic or α-neurotoxins have been reported to target muscle nAChRs with high affinity and selectivity; however, weak neurotoxins homologues (e.g., CM10, CM12 from *Naja haje annulifera* and S5C10 from *Dendroaspis jamesoni*), candoxin from *Bungarus candidus* and LSIII from *Laticauda semifasciata* have been demonstrated to produce a reversible neuromuscular blockade [20,21,22,23,24]. Similarly, individual conotoxins from the α3/5 and the α4/7 subfamilies, and the αA-family have shown remarkable selectivity, whereas ψ-conotoxins PIIIE and PIIIF have been reported as non-competitive antagonists for the muscle nAChRs [25,26,27].

In the present study, we assessed some *A. aurita* neurotoxic compounds on fetal and adult muscle nAChRs using the voltage clamp technique. The electrophysiological characterization was initially guided by the *in vivo* neurotoxic effects produced by a tentacle venom extract in crabs. The neurotoxic compounds were also tested in a qualitative enzymatic assay used as a preliminary indicator of phospholipase activity.

## 2. Results and Discussion

### 2.1. Jellyfish Specimens, Tentacle Venom Extract and Nematocyst Identification

*A. aurita* specimens were originally collected as polyps from coastal waters of the Gulf of Mexico (Veracruz, México) and subsequently raised to ephyras and adult jellyfish in the facilities of the marine park “Xcaret” (Quintana Roo, México). Specimens were identified as *A. aurita* species; however, due to the likely occurrence of cryptic species [28], the existence of more than one species of *Aurelia* in Mexican waters should not be discarded.

**Figure 1:**
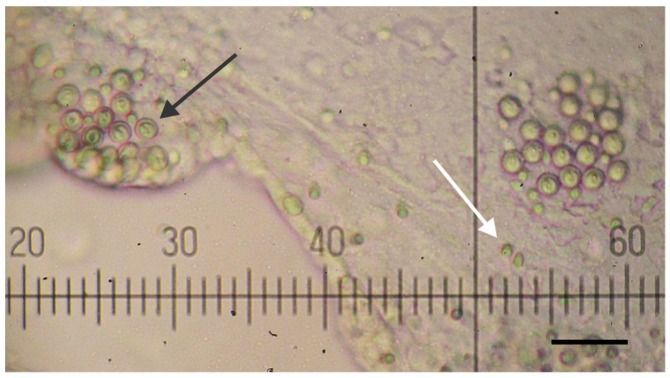
Micrograph of *A. aurita* nematocysts along fishing tentacles. Atrichous ishorizas (white arrow) measured 6 × 4 µm wide and presented a disperse distribution along the tentacle tissue. Heterotrichous microbasic euryteles (black arrow) were approximately 12 × 9 µm wide and were arranged in clusters. 400× magnification, bar = 50 µm.

Tentacle venom extract (TVE) was obtained from fishing tentacle preparations enriched with nematocyst batteries as shown in Figure 1. Two types of nematocysts were identified and corresponded to those previously reported [29,30]. Manual homogenization using a hand-held Teflon pestle and mortar was the most suitable technique for maximum nematocyst discharge (>80%) in comparison to methods like sonication or bead mill homogenization that caused massive rupture of capsules or complete destruction of the organelles. 

Although TVE contained nematocyst-derived venom, the presence of nematocyst capsule membranes and tissue components should be considered as a result of tentacle homogenization process. Therefore, the biological effects produced by this preparation should be attributed to all components within the extract and not only to nematocyst venomous contents. In this way, some bioactive compounds from other cnidarians have proven not to be exclusively confined to nematocyst structures but also found in other tissues like ectodermal gland cells or nematocyst-devoid tentacle extracts [31,32].

The biological activity of TVE was well preserved for almost one year using a protease inhibitor cocktail, lyophilization and storage at −70 °C. Similar preservation and concentration methods have been recommended for other jellyfish venom preparations by other investigators [33]. 

### 2.2. Protein Content Analysis and Fractionation

**Figure 2:**
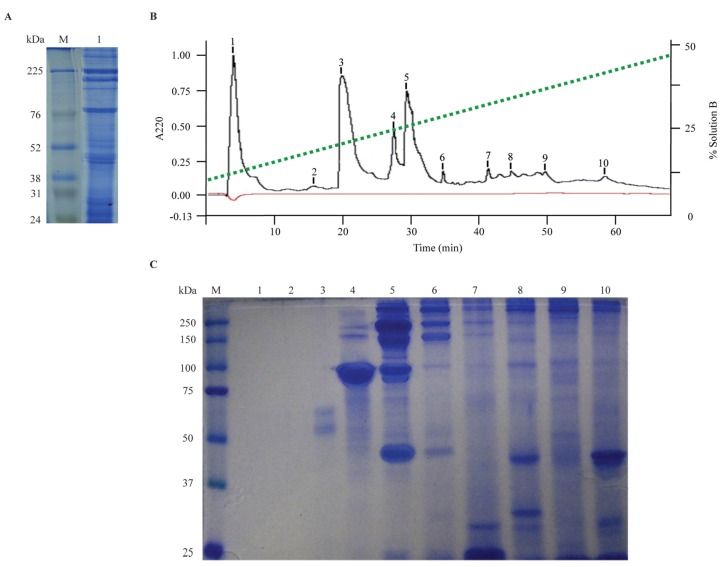
Protein content and fractionation of *A. aurita* TVE. (**A**) SDS-PAGE protein profile of TVE was performed in 12% polyacrylamide gel stained with Coomassie brilliant blue R-250. M indicates molecular mass standards (Amersham Rainbow Marker high-range, GE Healthcare); Lane 1 corresponds to 20 μg total protein of TVE. (**B**) Chromatogram of TVE fractionation by C18 reversed-phase column using a gradient of solution B from 5% to 95% (dashed line) at a flow rate of 1 mL/min over 60 min; Baseline indicated with a thin line. (**C**) SDS-PAGE protein profile of chromatography fractions. Analysis was performed in 10% polyacrylamide gels stained with Coomassie brilliant blue R-250. M indicates molecular mass standards (Kaleidoscope, Bio-Rad); Lanes 1–10 correspond to the eluted peaks equally labeled from HPLC fractionation.

The total protein yield of the TVE was 50 mg (dry weight) from 50 jellyfish. An initial overview of the *A. aurita* TVE protein profile was obtained by reducing SDS-PAGE (Figure 2A). A great number of proteins ranging from 24 to >225 kDa were detected, reflecting the complexity of TVE. The analysis revealed 6 major constituent proteins clearly-defined at 40, 80, 95, 100, 200, and 225 kDa, while diffuse protein bands were present at 24–31 kDa and 45–55 kDa. Similar results have been reported for this species elsewhere; however, the distribution of some protein bands varies among previous reports [7,8,12]. These differences have often been attributed to variability in isolation procedures, animal size, nematocyst source (e.g., oral arms or fishing tentacles), and geographical distribution of the species [12,34]. 

To further identify the protein compounds that induced neurotoxicity, TVE was fractionated using a C18 analytical reversed-phase HPLC column that yielded 10 fractions during the first 60 min of elution (Figure 2B). All chromatography fractions were also analyzed with SDS-PAGE which showed an evident separation of venom proteins (Figure 2C). For fraction 1 (50 µg apparent total protein) and 2 (4.5 µg apparent total protein) no protein bands were detected and may correspond to salts, unbounded compounds or trace contaminants. Only two poorly defined bands at ~55 and ~65 kDa were found in fraction 3 (3.6 µg total protein). Fraction 4 (28 µg total protein) was mainly composed of an intense band at ~90 kDa and two minor bands between 150 and 250 kDa. A less intense ~90 kDa band was also visible in fraction 5 (50 µg total protein), in addition to four more components detected at 45, 85, 150, 225, and >250 kDa. The profile of fraction 6 (37 µg total protein) showed protein bands at 150, 250 and >250 kDa, but the ~90 and ~45 kDa bands appeared more diffuse. Fractions 7, 8, 9 and 10 (25, 25, 39 and 0.8 µg total protein, respectively) revealed noteworthy protein bands in clusters at 25–35 kDa and 37–45 kDa, indicating lower molecular masses for the predominant protein components of these chromatography fractions, although some protein bands from 100 to 250 kDa are still distinguishable.

### 2.3. *In Vivo* Neurotoxic Effects

The identification of *A. aurita* neurotoxic TVE components was initially assessed *in vivo* by intramuscular injections into adult ghost crabs (*Ocypode quadrata*). Animals injected with control vehicle (deionized water) exhibited normal behavior. In contrast, intramuscular injections of *A. aurita* TVE (100 μg total protein) caused motor impairment, appendix stretching, tetanic reactions, loss of balance, flaccid paralysis, and death of crabs within 7 min. These neurotoxic reactions were similar to those reported initially by Segura-Puertas *et al.* [7], demonstrating that neurotoxicity is a consistent and clearly defined feature of *A. aurita* TVE. 

After fractionation, all resultant chromatography fractions were also tested in crabs. Fractions 4 and 5 (Figure 2B,C) were the only fractions to cause similar reactions to those of TVE. Fraction 4 (28 µg total protein) produced uncontrolled appendix movements and leg stretching reactions. These effects were temporary and crab recovered completely in 1 min. Fraction 5 (50 µg total protein) only caused temporary appendix movements for 20 s. 

### 2.4. *In Vitro* Characterization on Muscle nAChRs

**Figure 3:**
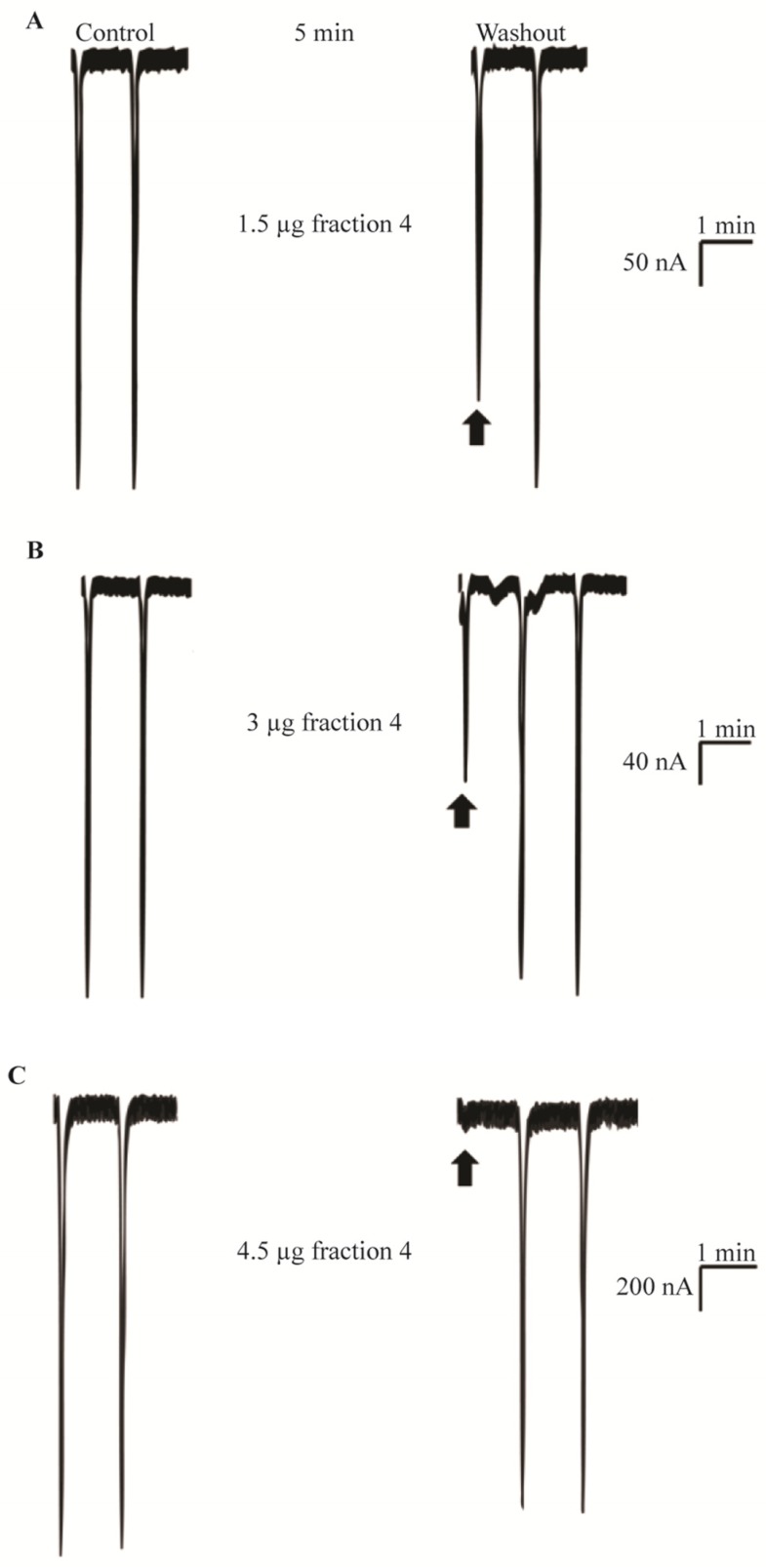
Activity of fraction 4 on fetal muscle nAChR expressed in *X. laevis* oocytes. Concentration-response effects can be compared through the various panels. Arrows indicate the first current elicited after the 5 min static bath of toxin equilibration. Control membrane depolarisations elicited by 1–5 μM ACh pulses are indicated as 10% of total value (nA); (**A**) 1.5 μg total protein of fraction 4 inhibited 25% of ACh-elicited currents; (**B**) 3 μg total protein of fraction 4 blocked 50% of ACh currents; (**C**) 4.5 μg total protein of fraction 4 caused 96% blocking effect.

Pulses of ACh applied to non-injected *X. laevis* oocytes showed the absence of native cationic channels in the oocyte batches used in this study. On the contrary, pulses of 1–5 μM ACh caused depolarization of transfected oocytes to 4–20 µA (details in Figure 3). 

**Figure 4:**
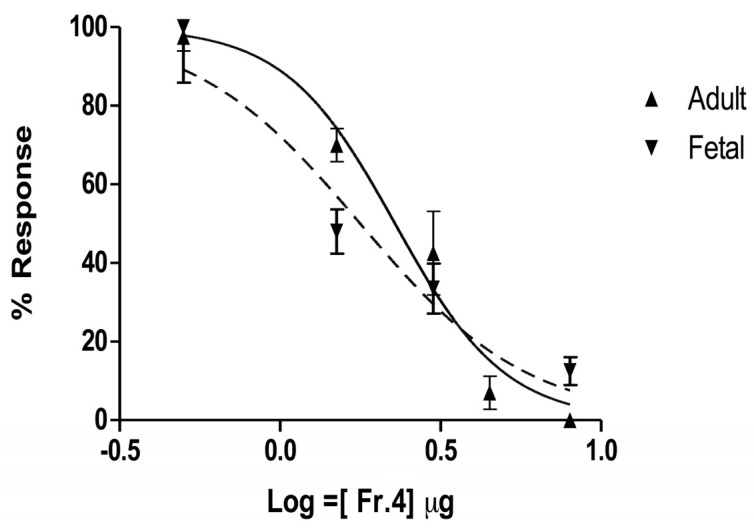
Concentration-response curves for fraction 4 on the adult (up-triangle) and fetal (down-triangle) subtypes of mouse muscle nAChR. Curves were generated by plotting current amplitude after toxin application as a percentage of current amplitude prior to toxin application (% response). Each data point represents the average value ± S.E. of the responses from three oocytes.

TVE and fractions 4 and 5 (Figure 2B,C) were tested on muscle nAChRs following the neurotoxicity exhibited *in vivo*. TVE produced membrane alterations and immediate death of oocytes even at low concentrations. Similarly, serial concentrations of fraction 5 caused damage to oocyte membranes preventing adequate current measurements. For this reason, TVE and fraction 5 were discarded for further *in vitro* tests. Fraction 4 potently inhibited ACh-elicited currents mediated by both fetal and adult muscle nAChR subtypes. The inhibitory effect for the fetal muscle receptors is shown in Figure 3. Receptor inhibition was concentration-dependent and completely reversible at the second pulse of ACh allowing complete toxin dissociation from receptors in all tested concentrations. The concentration-response curves indicate that fraction 4 had an IC_50_ value of 1.77 μg/μL and 2.28 μg/μL for fetal (α1β1γδ) and adult (α1β1εδ) muscle nAChRs, respectively (Figure 4). Although fraction 4 was not completely isolated, the main components of this fraction corresponded to a ~90 kDa protein.

The activity exhibited by fraction 4 showed a rapid reversibility similar to the temporary reactions observed in crabs demonstrating a correlation using *in vivo* and *in vitro* approaches. The toxin-receptor kinetics resembled those of *α*-neurotoxins, especially of non-conventional, three-fingered α-neurotoxins (e.g., candoxin and “weak toxins” also named WTX), as well as some members of the ψ-conotoxins (e.g., PIIIE and PIIIF) [23,24,35,36]. These neurotoxins exhibited similar dissociation kinetics (5 to 10 min to complete wash-out from receptors) and affected muscle nAChRs subtypes with different potencies. The fast dissociation could be ascribed either to low protein concentration or low receptor affinity; however, the protein concentration was enough to cause a blockade of muscle receptors as well as neurotoxic reactions in crabs. Furthermore, the toxin interaction with several nAChR subtypes is not always correlated with low receptor affinity and could reflect underlying mechanisms of interaction between toxin and receptor distinct from the specific binding sites [37]. Even though we were unable to specify the interaction site or mechanism of action of the partial purified fraction, toxin-receptor kinetics suggest a non-competitive antagonist activity. Nevertheless, further characterization of the receptor-toxin interaction remains to be carried out.

### 2.5. Phospholipase A Activity

Since it has been demonstrated that the venom of *Aurelia aurita* jellyfish contains phospholipase A_2_ activity [12], we investigated the presence of this activity in the neurotoxic fractions identified in this study (TVE and fractions 4 and 5). No cleared rings in the plates were visible with the negative control (deionized water). In comparison, the snake phospholipase used as a positive control and *A. aurita* TVE caused peripheral clear ring areas indicating a significant enzymatic activity. Fractions 4 and 5 did not cause cleared ring areas, thus demonstrating an absence of PLA activity (data not shown). This result suggests that neurotoxic fraction 4 lacks an enzymatic activity necessary to hydrolyze membrane phospholipids commonly shown by presynaptic neurotoxins [37,38]. Moreover, fraction 4 seems to have a preferentially postsynaptic action at the neuromuscular junction by inhibiting acetylcholine binding to postsynaptic nAChRs (Section 2.4), contrarily to the absent alteration of the motor endplate sensibility to acetylcholine exhibited by presynaptic neurotoxins [39]. In addition, fraction 4 produced flaccid paralysis on crabs when tested *in vivo* which is a common effect produced by some postsynaptic neurotoxins [39]. However, this hypothesis remains to be confirmed by biochemical and structural studies of the neurotoxic components of the bioactive fraction in order to clarify its mechanism of action. 

## 3. Experimental Section

### 3.1. Reagents

Protease inhibitor cocktail was purchased from Roche (Indianapolis, IN, USA), protein assay kit (DC™) was obtained from Bio-Rad (Hercules, CA, USA), gentamicin was from Bruluart (Naucalpan de Juárez, México) and all other chemicals of analytical grade were from Sigma (St. Louis, MO, USA). Solvents were high performance liquid chromatography (HPLC) grade.

### 3.2. Specimen Collection

*A. aurita* polyps were collected from the Gulf of Mexico (Veracruz, México) and cultured in the facilities of the marine aquatic park “Xcaret” (Quintana Roo, México) in April 2010. A total of 50 adult specimens were acquired. Live jellyfish were individually placed in 1-liter containers filled with ice-cold seawater at 4 °C and immediately transported to the laboratory. 

### 3.3. Preparation of Tentacle Venom Extract

*A. aurita* tentacle venom extract (TVE) was obtained according to Segura-Puertas and colleagues [7]. Briefly, fishing tentacles were excised, combined and placed in cold deionized water at 1:4 *v*/*v* with a tablet of protease inhibitor cocktail (one tablet for 50 mL) (Complete, MiniProtease Inhibitor Cocktail, Roche, Indianapolis, IN, USA). Tentacles were homogenized using a hand-held Teflon pestle and mortar (Pyrex^®^ 40 mL Ten Broeck Homogenizer, Palo Alto, CA, USA) surrounded by an ice slurry. Nematocyst discharge was monitored microscopically until maximum capsule discharge was achieved. The mixture was then centrifuged at 18,700 × *g* for 10 min at 4 °C (Eppendorf centrifuge, swinging bucket rotor, Hamburg, Germany). The supernatant was collected, lyophilized (Savant SpeedVac drier, Waltham, MA, USA), and separated into aliquots of 1 mg dry weight. Lyophilized TVE was stored at −70 °C. Identification of discharged and undischarged nematocysts was based on capsule and tubule characteristics according to the Östman [40] nomenclature guideline and previous reports.

### 3.4. Chromatography Fractionation of Tentacle Venom Extract

For each chromatography run, an aliquot of lyophilized TVE (1 mg total protein) was dissolved in 1 mL of 5% *v*/*v* aqueous acetonitrile (ACN) containing 0.004% *v*/*v* trifluoroacetic acid (TFA). Dissolved TVE was applied to a Vydac C18 analytical reversed-phase HPLC column (218TP54, 5 μm, 4.6 × 250 mm, 300 Å) equipped with a Vydac C18 guard column (218GK54, 5 μm, 4.6 × 10 mm). Protein elution was accomplished using an HPLC chromatography system (Pro Star, Varian, Palo Alto, CA, USA) with a linear gradient of 5%–95% solution B at a flow rate of 1 mL/min over 90 min. Solution A was 0.1% *v*/*v* aqueous TFA and solution B was 0.085% *v*/*v* TFA in 90% *v*/*v* aqueous ACN. The absorbance of the eluate was measured at 220 nm. The resultant chromatography fractions were collected manually in 1.5 mL polypropylene tubes, freeze dried (Savant SpeedVac drier, Waltham, MA, USA), and stored at −70 °C.

### 3.5. Protein Concentration

The protein concentration of TVE and chromatography fractions were determined in a microplate reader spectrophotometer using a commercial kit assay (DC™ Protein Assay, Bio-Rad, Hercules, CA, USA) with bovine serum albumin (BSA) as a reference standard.

### 3.6. SDS-PAGE Analysis

Protein profiles of TVE and chromatography fractions were obtained by sodium dodecyl sulphate polyacrylamide gel electrophoresis (SDS-PAGE) [41]. An aliquot of TVE (1 mg total protein) and lyophilized chromatography fractions (0.8–50 µg total protein) were individually dissolved in 10 µL of deionized water and mixed with 10 µL of reducing sample buffer. Protein samples were boiled for 4 min and loaded into the lanes of electrophoresis gels. The one-dimensional electrophoresis was performed in 10% and 12% polyacrylamide separating gels using a Bio-Rad Mini-PROTEAN 3 electrophoresis system. Gels were run at 60 V for 4 h in Tris-glycine buffer, pH 8.3, at 4 °C. Protein bands were visualized with Coomassie brilliant blue R-250 (Sigma-Aldrich, St. Louis, MO, USA) and molecular masses were estimated by comparison with 10–250 kDa protein standards (Kaleidoscope, Bio-Rad; Amersham Rainbow Marker high-range, GE Healthcare, Hercules, CA, USA).

### 3.7. Neurotoxic Activity Bioassay

The neurotoxic activity of TVE and chromatography fractions was assessed by a crustacean bioassay used to characterize cnidarian neurotoxins [42,43]. In brief, male adult ghost crabs (*Ocypode quadrata*) with 10–15 g total body weight were collected from the Caribbean coast of Quintana Roo, México and immediately used in the bioassay. All crabs were injected intramuscularly into the base of the third walking leg using 29-gauge hypodermic needles. Test crabs (*n* = 3) were injected with TVE (100 µg total protein) or lyophilized chromatography fractions (0.8–50 µg total protein) dissolved in 0.2 mL of deionized water. Control crabs (*n* = 3) were injected only with 0.2 mL of deionized water. Behavior after injection was observed simultaneously in treated and control crabs. All experimental procedures in animals were approved by the Mexican official norm for the use and care of laboratory animals “NOM-062-ZOO-1999”.

### 3.8. Activity on Nicotinic Acetylcholine Receptors

Electrophysiology was performed as described in detail previously by Cartier *et al.* [44]. Briefly, oocytes isolated from *Xenopus laevis* frogs were defolliculated with 1 mg/mL of collagenase (Collagenase IA from *Clostridium histolyticum*, Sigma, St. Louis, MO, USA) dissolved in OR2 solution in gentle shaking for 45 min. OR_2_ solution consisted of 82 mM NaCl, 2 mM KCl_2_, 1 mM MgCl_2_, and 1.8 mM HEPES, pH 7.5. Defolliculated oocytes were then washed with OR_2_ solution alone and placed in ND96 solution with 125 µg/mL gentamycin (Bruluart, Naucalpan de Juárez, México) at 17 °C. The composition of the ND96 electrophysiological solution was: NaCl 96 mM, KCl 2 mM, CaCl_2_ 1.8 mM, MgCl_2_ 1 mM, HEPES 5 mM, pH 7.5. 

Oocytes were transfected 1–2 days after harvesting. Plasmid DNA was cloned in cytomegalovirus (CMV)-based pRBG4 vector and encoded for mouse muscle nAChR subunits [45]. 1 ng of each subunit cDNA (α1, β1, ε, δ, γ) was injected into the nucleus of each oocyte to express fetal (α1β1γδ) or adult (α1β1εδ) muscle nAChR subtypes. Non-injected oocytes were used as negative controls for nAChR subtype expression.

Voltage-clamp recordings were made 1–6 days after injection. Each oocyte was placed in a 30-μL recording chamber, clamped at −70 mV with two electrodes, and gravity perfused with ND96 solution. Acetylcholine (ACh)-gated currents were elicited by 1 s pulse/min of 1–5 μM ACh. MlatA1 from *Micrurus laticollaris* was used as muscle nAChR antagonist [46]. 

Electrophysiological characterization was conducted only on TVE and chromatography fractions that exerted neurotoxicity in the crab bioassay as previously described. TVE (100 µg total protein) and neurotoxic fractions (0.5–60 µg total protein) were dissolved in 18 μL of ND96 solution and serial dilutions were evaluated by application in the superfusing bath. A series of three-way solenoid valves (Neptune Research, Northboro, MA, USA) were used to switch the perfusion medium between ND96, toxin or ACh. TVE and neurotoxic fractions were allowed to equilibrate with the receptors in a static bath for 5 min before pulsing with ACh. Subsequently, ND96 perfusion and ACh pulses were resumed to permit toxin dissociation from the receptors and washout. For control responses, ND96 solution alone was applied in a 5 min static bath prior to pulsing with ACh.

Test and control responses were assessed in at least 3 different oocytes. The average peak amplitude of the control responses just preceding the exposure to toxins was used to normalize the amplitude of each test response to obtain a “% response” or “% block”. Concentration-response curves were fitted to the equation: % response = 100/(1 + (toxin/IC_50_) *n*H), where *n*H is the Hill coefficient, using Prism software (GraphPad Software Inc, San Diego, CA, USA). Each point represents the average value ± S.E. of the responses of the tested oocytes. All electrophysiological experiments were performed using an oocyte clamp system (Warner Instruments Corp., Hamden, CT, USA; Model OC-725C) at room temperature.

### 3.9. Phospholipase A (PLA) Assay

The PLA enzymatic activity was assessed by a simple and highly sensitive radial diffusion assay using a modification of the Habermann and Hardt method [47]. Only the neurotoxic fractions of *A. aurita* (TVE and chromatography fractions 4 and 5) were tested in this assay. In brief, a mixture of 1 mL of 20 mM CaCl_2_, 100 µL of Triton X-100 and 2 mL of egg yolk suspension (30%) was added to 10 mL of 0.6% agarose dissolved in 0.2 M Tris-HCl buffer, pH 7.95. The solution was poured into plastic Petri dishes and dried at 37 °C. TVE and neurotoxic fractions (50 μg total protein) were dissolved in 5 µL of deionized water and placed into wells. Plates were incubated for 24 h at 28 °C. PLA activity was detected visually by peripheral clear ring areas surrounding wells. A purified phospholipase A_2_ from an elapid snake (*Micrurus tener*) was used as positive control (data unpublished) and deionized water was used as negative control. Comparison of clear haloes was performed using UV transillumination (Gel Logic 200 Imaging System, Kodak, Rochester, NY, USA).

## 4. Conclusions

Tentacle venom extracts obtained from *A. aurita* jellyfish cause well-defined neurotoxic effects when tested *in vivo*. However, the mechanisms of action underlying this noxious process have remained unclear. The identification of possible molecular targets by means of electrophysiological analysis can lead to a better understanding of the interaction and the molecular mechanisms involved. Here, we described for the first time the interaction between some components of a tentacle venom extract from *A. aurita* and muscle nicotinic acetylcholine receptors. The neuromuscular effects produced by some venom components suggested the participation of receptors located at the neuromuscular junction, which activity was confirmed and correlated *in vivo* and *in vitro*. Moreover, the neurotoxic fraction lacked phospholipase A activity, thus suggesting a preferential interaction with postsynaptic receptors such as muscle nicotinic acetylcholine receptors. These results may explain, at least in part, the neurotoxicity and paralysis observed in crabs, but also the effect seen on nicotinic receptors agrees with the evidence that *A. aurita* might feed of juvenile crustaceans found in the zooplankton as a part of its diet. 

Further biochemical studies will allow the identification and analysis of composition and structural features of protein compounds implicated in neurotoxicity. Electrophysiological and pharmacological characterizations are also needed to determine the specificity and selectivity of scyphozoan neurotoxins on other membrane receptors or ion channels.
